# Exploring imaged capillary isoelectric focusing parameters for enhanced charge variants quality control

**DOI:** 10.3389/fchem.2025.1536222

**Published:** 2025-02-20

**Authors:** Virginia Ghizzani, Alessandro Ascione, Federico Gonnella, Gabriella Massolini, Francesca Luciani

**Affiliations:** ^1^ National Centre for the Control and Evaluation of Medicines (CNCF), Istituto Superiore di Sanità, Rome, Italy; ^2^ Department of Drug Sciences, University of Pavia, Pavia, Italy

**Keywords:** icIEF, charge heterogeneities, isoelectric point, biotherapeutics development, QC

## Abstract

Biopharmaceuticals are increasingly utilised in the treatment of oncological, inflammatory, and autoimmune diseases, largely due to their exceptional specificity in targeting antigens. However, their structural complexity, heterogeneity, and sensitivity pose crucial challenges in their production, purification, and delivery. Charge heterogeneity analysis, a Critical Quality Attribute of these biomolecules used in their Quality Control, is often performed using separative analytical techniques such as imaged capillary Isoelectric Focusing (icIEF). Recognized as a gold standard by the industry, icIEF leverages a pH gradient to provide high-resolution profiling of charge variants in biotherapeutics. In this review, critical experimental parameters for icIEF method development in the context of biotherapeutic drug development and QC will be discussed. Key aspects, including sample preparation, capillary properties, carrier ampholytes, stabilizers, and detection are examined, and supported by recent literature. Advances in icIEF technology and its expanding applications underline its robustness, reproducibility, and compliance with regulatory standards, affirming its pivotal role in ensuring the identity and consistency of biological products.

## 1 Introduction

Biotherapeutics are becoming commonly used drugs for the treatment of several oncological, inflammatory, and autoimmune diseases principally due to their high specificity in target antigen binding, reducing the need for frequent dosing (Lechner et al., 2019, 1–17; Sharma et al., 2023, 18; Paul et al., 2024, 399). Many of the biotherapeutic molecules are monoclonal antibodies (mAbs), however, recently, several novel antibody-based biologic products have been engineered in order to improve potency, increase circulation half-life, expand functions, enable specific delivery of drugs and effector proteins to the site of action, and enhance tissue penetration. Examples of next-generation of antibody therapeutics include Fc fusion proteins, antibody-drug conjugates (ADC), bispecific antibodies (BsAbs) and antibody fragments. Today, more than 100 mAbs and ADCs have been approved as biotherapeutic products by the European Medicines Agency (EMA) and the Food and Drug Administration (FDA) (Carter and Rajpal, 2022, 2789). These biomolecules are often complex, heterogeneous, and fragile, which makes their production, purification, and delivery challenging. Indeed, during their production in cell culture and storage, biotherapeutics are prone to Post-Translational Modifications (PTMs) such as deamidation, glycosylation, or oxidation which may produce charge heterogeneities. Since some charged-based variants can have an impact on pharmacokinetics, biological activity, and long-term storage, charge heterogeneity is considered a Critical Quality Attribute (CQA) by regulatory authorities which must be monitored during the biotherapeutics life cycle, to ensure their quality, efficacy, and safety. In fact, a CQA is a physical, chemical, biological, or microbiological property/characteristic that should be within an appropriate limit, range, or distribution to ensure the desired product quality (Pharmaceutical Development, 2022, ICH Harmonised Tripartite Guideline). CQAs variation, outside defined ranges, can have an impact on the final drug product (DP) safety and efficacy.

Quality Control (QC) of charge heterogeneity is commonly achieved using separative analytical techniques such as ion exchange chromatography (IEC) or traditional isoelectric focusing (IEF), particularly conventional capillary isoelectric focusing (cIEF) or imaged capillary isoelectric focusing (icIEF) (Lechner et al., 2019, 1–17)**.** icIEF combines the principles of capillary electrophoresis (CE) and IEF to separate proteins based on the isoelectric point (pI) within a capillary, the separation of analytes occurs along the length of the capillary by direct imaging with a charge-coupled device (CCD) camera, without a mobilisation step as in conventional cIEF (Zhang et al., 2016, 148; Madren et al., 2022, 1050)**.**

Compared to cIEF, icIEF allows faster separation, higher resolution, repeatability, and a simpler method development procedure. Moreover, it can be applied to biopharmaceutical QC due to its good sensitivity and robustness, thus being a useful tool to be used for annual marketing surveillance programs and the fight against counterfeit drugs. The icIEF protocols can be efficiently validated according to ICH guidelines. These advantages offer in the regulatory context a potential analytical platform for an effective detection of several PTMs-related charge-isoforms.

**FIGURE 1:**
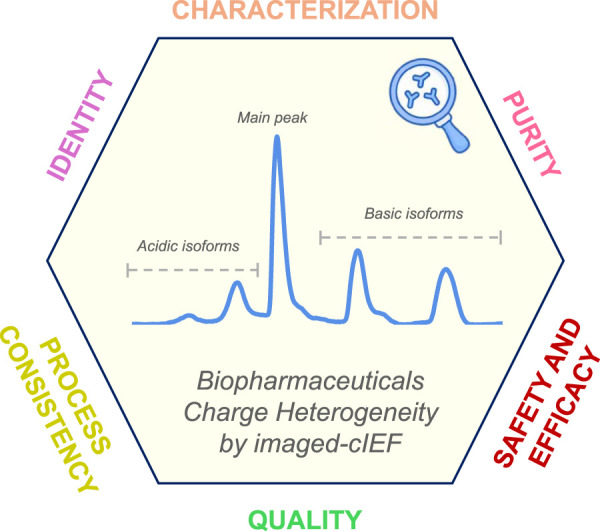
Illustrative schematic diagram showing the valuable insights obtained with icIEF charge variants analysis that support the characterization, identification, purity assessment, and process consistency of biotherapeutics. These attributes are critical for evaluating the quality, efficacy, and safety of drug products, thereby contributing significantly to their development and QC.

Recently, thanks to the increasing use of (i)cIEF, European Pharmacopoeia (Ph. Eur.) published a general text in which it is proposed a horizontal method to analyse mAb-based drugs and their charge variants, making appropriate technical distinctions between the classical and the imaged method (European Pharmacopoeia, 2023). In this framework, some interesting reviews have been published. Kahle and Wätzig have discussed the application of three electrophoretic based techniques, namely, cIEF, icIEF, and capillary zone electrophoresis for the analyses of protein charge variants (Kahle and Wätzig, 2018, 2492)**.** The article defined the experimental conditions in icIEF methods development, such as concentration of carrier ampholytes (CAs), L-Arginine (L-Arg), pI Markers (pIMs), and urea, in QC of mAbs. More recently, Krebs et al. published a review on CE method development and validation including icIEF, emphasising that this is a mature technique that can be routinely applied in analytical laboratories, especially in the biopharmaceutical industry (Krebs et al., 2023, 1279)**.** Another excellent review has been published in Trends in Analytical Chemistry mainly focused on the current developments of icIEF technologies including higher sensitivity detection mode (fluorescence and chemiluminescence) and combination of icIEF with mass spectrometry (MS) detection. Applications of icIEF in the pharmaceutical industry and in research laboratories were also discussed by Wu (Wu J. et al., 2022, 150)**.** In the current state of the art, icIEF is an elected analytical method for determining pIs and charge heterogeneity profiles to guarantee the QC during the entire life cycle for mABs, BsAb, ADCs and biotherapeutic proteins (Kinoshita et al., 2013, 76; Tardif et al., 2023, 124633; Sutton et al., 2024, 5450–5458; Wu et al., 2024a) (Figure 1). In literature are reported different validation studies and QC methods for biotherapeutics published by industry users and researchers of icIEF technology (Wu et al., 2018, 2091–2098; Li et al., 2020, 3836–3843). For mAbs, icIEF methods are typically used as identity and/or purity assays in the pharmaceutical industry. The uniqueness of the charge heterogeneity profile of a mAb product is used for identity, commonly coupled to other analytical techniques, such as peptide mapping or bioassays. In the purity assay, the pI value and percentage of each charge variant of a mAb are determined. The combination of the two values of all peaks is the charge profile of the mAb, which is often used in formulation studies and product QC (Wu et al., 2024a). Product identity is one of the release testing requirements that need to be established to ensure no misidentification of drugs.

In this review, we will focus on the icIEF advancements and experimental conditions to be considered during method development for biotherapeutics drug development and QC. An overview of major performance parameters, such as sample preparation and additives, capillary properties, CAs, anodic and cathodic stabilizers, and detection modes will be discussed with the support of well-established literature and articles of the last decade. The developments of this technology and the more novel applications indicate that icIEF is a robust, reproducible, and regulatory-compliant method for ensuring the identity and consistency of biological products (Ahluwalia et al., 2018, 271).

## 2 icIEF technique

icIEF is an industry’s high-resolution gold-standard separative technique based on a pH gradient and is employed for the evaluation of charge variants profiles of biotherapeutic (Madren et al., 2022, 1050–1058)**.** It can be considered the latest evolution of the outdated IEF separation technique, a group of analytic methodologies that operates in the presence of an electric field and has emerged as a tool for detailed charge-based analysis of complex molecules, especially biological drugs. Similarly to the other IEF techniques, it exploits the properties of ampholytic components, which are molecules acting as weak acids and bases, to separate them in the presence of an electric field. The electrophoretic mobility of these species will change in the presence of a pH gradient, slowing migration in the region near their pI value, by definition the pH level where the net charge of the species is zero. In the case of protein molecules, the process of separation is based on the composition of the exposed amino acids and charged residues, which behave in a manner analogous to weak acids and bases. It is crucial to acknowledge that the pI value determined through (i)cIEF techniques is regarded as an “apparent” pI, since experimental conditions influence it. This distinction stems from the observed divergence between experimentally measured values and theoretical predictions, typically derived from the primary sequence of the molecule (Ascione et al., 2024, 2313737).

**FIGURE 2:**
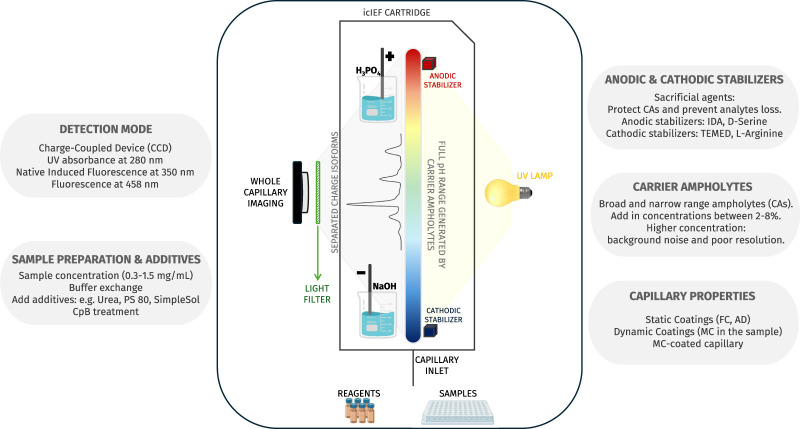
The figure provides a detailed scheme of icIEF technology which is able to detect the entire capillary, thereby allowing real-time monitoring of analyte charge variants separation. Additionally, the figure gives an overview of the factors that influence the method’s performance.

While the initial application of this separative approach was the polyacrylamide gel-based IEF (gIEF), the cIEF was first developed in the early 1980s thanks to the pioneering work of Hjertedal and Zhu (Hjertén and Zhu, 1985, 265–270)**.** Further relevant work was given, for instance, by Chen A. B.'s research group (Jochheim et al., 2001, 59–65). However, the earliest iteration of icIEF occurred approximately a decade later, in the early 1990s, with the progressive work of Wu and Pawliszyn, (1994) research group, who innovated the method by introducing whole capillary imaging (real-time monitoring of the separation phase) instead of a single point of detection (Wu and Pawliszyn, 1992, 219–224, 1994, 867–873). However, similarly to all related IEF techniques, multiple parameters must be considered in order to ensure the success of the method, as will be discussed in the relevant sections of this review (Figure 2; Table 1).

**TABLE 1:** Summary of main critical aspects to be addressed during an icIEF method development.

Factor	Parameter	Aim	Influence on the analysis	Drawbacks
Capillary coatings	Fluorocarbon (FC)	Suppressed EOF	Improve resolution and peak shape	
	Acrylamide (AD)	Reduced EOF Reduced adsorption of biomolecules		
	Methylcellulose (MC)	Reduced EOF		MC not compatible with MS analysis, could produce spikes and clogs
Sample preparation	Concentration (not < 0.2 mg/mL)	Experimental setting based on LOD_ABS_ and LOD_FLUO_	Suitable sensitivity	
	Buffer Exchange	Removing interferences from sample matrix	Reduced spikes and artefacts	
	Additives (Urea, NDSB, Glycine, SimpleSol)	Enhanced solubility, avoid precipitation and aggregation	Repeatability, reliability, reproducibility	Could produce peak shifts and spikes
Carrier ampholytes	Broad-range	pH gradient more versatile and widely utilised		
	Narrow-range	Tailored analysis for specific biomolecules	Enhanced resolution	Longer focusing time needed
	Total concentration (ranging 2%-8%)	Method optimization	Enhanced separation	>4 % could increase background noise
Anodic and cathodic stabilizers	Highly acidic pI value (IDA, Serine-D)	Reduced anodic drift	Stable pH gradient, enhanced repeatability, and reproducibility	High concentration negatively affect resolution
	Highly basic pI value (L-Arg, TEMED)	Reduced cathodic drift		
Focusing time	Sample-tailored (ranging 4-15 minutes)	Method optimization	Enhanced separation	Too short time: incomplete focusing. Too long time: not reliable pI values, lost pI Markers and/or analytes, artefacts and spikes
Detection mode	Absorbance	Signal acquisition tuning	Standard detection	Higher background noise
	Fluorescence		Higher sensitivity and resolution, decreased need of buffer exchange, spikes recognition	More limited available type of pI Markers

Technically in icIEF, as in cIEF, a designed mixture of amphoteric molecules, called CAs, is used to generate a pH gradient when an electric field is applied between the end of the capillary, which enables the analytes to move along the capillary until they reach their pI value. Within this context, the focusing time constitutes another pivotal parameter that must be methodically delineated during the method development to ensure the attainment of an optimal separation and, concomitantly, a reliable estimate of the measured pI values. Insufficient focusing time may result in some charged species not reaching their isoelectric point, thus leading to incomplete separation. Conversely, excessive focusing time may cause a shift in the peaks due to undesirable factors such as a residual effect of the EOF or the secondary occurring of pH gradient instability.

Considering its fundamental principles, it is evident that the genesis and development of reliable ampholyte formulations to generate stable pH gradients has been a pivotal aspect in the evolution and application of this separative technique (Righetti et al., 1997, 91–104). The implementation of the methodology within the capillary framework has been demonstrated to result in a notable enhancement in the resolution that permits differentiation between the most closely related charge variants, accompanied by a considerable reduction in both focusing time and the amount of samples required (by order of few μL), which are considered advantageous aspects for the routine monitoring of pharmaceutical products. While these significant advantages are shared between cIEF and icIEF, the latter actually represents a further evolution of the conventional one with additional advantages, notably in terms of automation and faster analysis. In fact, this improvement renders the mobilisation step unnecessary, which is instead a prerequisite for conventional cIEF in order to push the analytes, once separated, towards the detector (for details, please see section 2.5).

Furthermore, icIEF technique can guarantee adequate sensitivity, enabling the detection of low abundance isoforms, with limit of detections (LODs) between 3 μg/mL and 0.7 μg/mL, depending on the detection mode (absorbance or fluorescence), as reported by the device manufacturer (Improving Charge Variant, 2000).

The icIEF has also proven to offer an optimal approach for the relative quantification of individual species (percentage area) or absolute levels, over the primordial gIEF technology, provided that adequate standards are available (Sosic et al., 2008, 4368).

As a consequence of the aforementioned advantages, icIEF is currently one of the leading methods in the industry for the analysis of biotherapeutics charge variants (Zhang et al., 2017b, 1–7). The technology of icIEF instruments has improved over the years and icIEF is now an indispensable tool in therapeutic protein development and manufacturing. The robustness and reproducibility of an icIEF instrument (iCE280) has been first evaluated in intercompany studies (Salas-solano et al., 2012, 3124) more than 10 years ago. The results from this study, whose statistical analysis was performed based on the ISO 5725-2 Guide principles, showed that icIEF is a reliable technology and largely met industry standards to assess charge heterogeneity of therapeutic proteins. Nevertheless, validation of the icIEF following the guidelines established by the International Council for Harmonization (ICH) remains to be addressed. In 2018, an interlaboratory icIEF method validation, involving 10 laboratories in eight independent Chinese companies using iCE3 instrument with improvements in the autosampler and injector, was carried out. The method validation was performed following the ICH guideline on the analytical procedure (specificity, precision, accuracy, linearity, range, limit of quantification (LOQ), and robustness) using a typical therapeutic mAb, with a single main peak at a pI of approximately 8.5. Taking all parameters together, the obtained results validate the use of the icIEF methodology as both an identity assay and purity assay in protein charge characterization (Wu et al., 2018, 2091–2098).

More recently, additional improvements to the icIEF equipment have resulted in the Maurice^®^ instrument by ProteinSimple, which utilises a pre-assembled cartridge leading to reduced instrument setup time. In an interesting application note, two different icIEF instruments, iCE3 system and the latest version Maurice^®^, were compared to understand if both equipment give comparable responses in terms of percentage of mAbs charge isoforms. A method validation was performed to estimate both method performances, concerning precision and LOQ and it has been concluded that both instruments display comparable performance in charge isoforms characterization of mAbs (Tavernier et al., 2022).

To demonstrate the comparability between iCE3 and Maurice, a global multi-lab study was carried out with a team of 19 companies (biopharmaceutical companies, diagnostics companies, and regulatory agencies located in the United States, Europe, and China). NISTmAb reference material and a programmed death-ligand 1 (PD-L1) fusion protein were analysed using both the iCE3 and Maurice instruments. Intra- and interlaboratory precision and robustness of the icIEF method for the two different molecules on both instruments were evaluated. The obtained results showed that both the iCE3 and the Maurice systems can robustly perform icIEF to monitor charge heterogeneity of monoclonal antibodies and fusion proteins. The electropherograms of the NISTmAb and the rhPD-L1-Fc are consistent across all laboratories and between both the two instruments. Identical apparent pI values (RSD values of less than 0.3% on both instruments) for the main isoform and comparable relative peak areas for the acidic, main, and basic isoforms for the NISTmAb were found (charged variants percent peak area values for both instruments less than 1.02% across different laboratories). Both instruments produce comparable quantitative results for rhPD-L1-Fc (Madren et al., 2022, 1050–1058).

**FIGURE 3:**
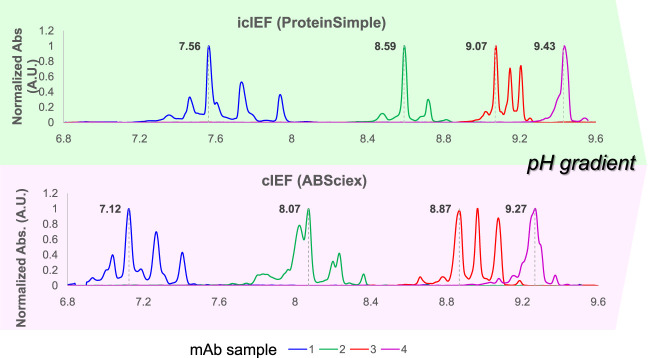
Systematic inconsistencies in measured pIs and charge distribution profiles have been observed when comparing cIEF and icIEF techniques across a selection of four mAbs with pI values ranging from 6.8 to 10. This set of therapeutic mAbs was analysed under similar experimental conditions. The electropherograms, obtained respectively by icIEF and cIEF (ProteinSimple and ABSciex), according to their own calibration curves, were scaled on the same pH range (normalized ABS signal vs. pI), to obtain comparable results. Reproduced and modified with permission from the ref. (Ascione et al., 2024, 2313737), Copyright 2024, Taylor and Francis Group, LLC. (This work is licensed under CC BY-NC-ND 4.0. To view a copy of this license, visit https://creativecommons.org/licenses/by-nc-nd/4.0/).

From an alternative perspective, Ascione et al. recently documented and discussed the parallel analysis of a panel of antibody products employing cIEF and icIEF systems, performed within the framework of the Ph. Eur. activities concerning the development of ‘horizontal standards’ for the QC of mAbs. This work showed that, despite the utilisation of comparable experimental conditions, inconsistencies emerge in the measured charge profile and isoelectric points between the two (i)cIEF systems, as shown in Figure 3. The reasons for this discrepancy are thought to be due to intrinsic differences between the instrumentations or to the commonly accepted practice of using too few pIMs as internal calibrators, leading to small deviations due to the assumption of linearity of the pH gradient along the capillary. As a consequence, it was concluded that, currently, cIEF and icIEF may not be considered directly interchangeable, and presented a thoughtful analysis of the implications of this from both an analytical and a normative perspective (Ascione et al., 2024, 2313737).

### 2.1 Capillary properties

Fused-silica capillaries used in conventional cIEF have silanol groups whose pK varies between pH 3.5 and 8. These slightly acidic groups generate a double layer at the capillary wall that plays an important role in developing the electroosmotic flow (EOF) (Yao et al., 1993, 21–29)**.** Large proteins can contain positive charge regions that are electrostatically attracted to the negatively charged silanol groups on the capillary inner surface. During icIEF separation, the EOF generated along the capillary by the adsorption may interfere with the separation of proteins according to their pIs (Štěpánová and Kašička, 2022, p. 339447; Duša et al., 2023, p. 117018). Therefore, neutral coating technologies based on hydrophobic fluorocarbon (FC) or hydrophilic acrylamide (AD), both of which are chemically linked to the capillary wall (static coating), have been widely employed to suppress EOF and enhance the efficiency of protein separation by icIEF (Kwok et al., 2023a, no. 5). However, the residual negatively charged silanol groups, which the coatings cannot fully shield, tend to adsorb the proteins, resulting in low separation efficiency and poor repeatability. Neutral polymers such as methylcellulose (MC) are usually added to the protein sample solution containing proteins and ampholytes to create a dynamic coating to improve the resolution and peak shape, especially for complex proteins (Kwok et al., 2023b, no. 5). The use of MC has some drawbacks such as the production of spikes due to bubble generation and frequent capillary clogs during the separation. Moreover, MC easily produces the contamination of MS ion source when carrying out icIEF-MS direct coupling (Permanent methylcellulose, 2023, coated cartridges achieves iCIEF free from polymers as dynamic coating for straight forward characterization of protein drugs - Technical note, Advanced Electrophoresis solutions). In a research article by Kwok et al., a bilayer polymerization strategy was developed for the static coating of MC in the capillary and the MC-coated capillary was employed to analyse charge variants of different types of complex biotherapeutics. It was observed that the peaks of the fusion protein were acidic (range of pI 4.0-6.5), the BsAb demonstrated rather basic pIs (around 9.5) for the main protein and its four isoforms, lastly the ADC sample showed basic properties (range of pIs 8.7–9.2) for major peaks. The new icIEF method demonstrated high repeatability, outstanding separation efficiency, and excellent pI measurements. The removal of MC from the experimental workflow greatly improved the compatibility with MS; thus, the MC-coated capillary was successfully used for the icIEF-MS characterization of protein charge variants for a diverse set of protein therapeutics (Kwok et al., 2022, 2).

### 2.2 Sample preparation and additives

In the analysis of biopharmaceutical products, sample preparation is an important part of icIEF method development. For example, mAbs formulations can contain high concentrations (10–100 g/L) of the mAbs and relatively high concentrations of buffers, salts, and excipients (e.g., sucrose, polysorbate 80), thus the samples must be appropriately processed in order to minimise the influence of concentration and excipients on the performances.

Depending on the initial concentration of the biomolecule to be analysed, the sample concentration should be adjusted taking into account the sensitivity of the instrumentation (as declared by the manufacturer) as well as the LOD/LOQ of the specific employed analytical method. Methods like dialysis or centrifugal filtration (using filters with a molecular weight cut-off) are commonly exploited to exchange the sample buffer in order to avoid unwanted matrix effect. The second approach can also be used, if necessary, to change the protein concentration to make it suitable for icIEF analysis. The sample buffer used must be chosen carefully to ensure that the pH gradient established along the capillary ensures a stable pI value. Typically, the buffers used are weak acidic or near neutral with low ionic strength, to avoid a denaturing process. Buffer exchange is considered a critical step to remove matrix components (Dadouch et al., 2021, 4) which might interfere with the icIEF analysis. In this regard, even the general chapter published in the Ph. Eur. on (i)cIEF analysis for recombinant therapeutic mAbs, provides a general recommendation to carry out a desalting step (using a common 20 mM Tris buffer at pH 8.0), aiming at removing any possible interferences related to the original formulation components of the analyte (2.5.44. Capillary Isoelectric Focusing for Recombinant Therapeutic Monoclonal Antibodies, 2023).

In a study by Abbood, the effects of varying the final concentration of maytansinoid-antibody samples (ADC) on charge variant separation were investigated (Abbood, 2023, 8150143). The samples were examined at 0.3, 0.5, 0.8, 1, and 1.5 mg/mL, and a shift to higher pI values was observed at higher concentrations. This phenomenon is likely attributable to the increased presence of auxiliary components, such as histidine, sucrose, and glycine, within the sample formulation, which may influence the linearity of the pH gradient. The optimal pH gradient linearity was observed within the concentration range of 0.3–1 mg/mL of maytansinoid-antibody. In a research article by Tardif et al., a Principal Component Analysis (PCA) was employed to evaluate the potential impact of sample concentration and excipients on the electrophoretic profile that could serve as a fingerprint for unambiguous analytes (mAbs) identification (Tardif et al., 2023, 124633). Infliximab was diluted with ultrapure water or Polysorbate 80 (PS 80). Higher quantities of PS 80 were selected in comparison to those typically employed in hospital settings (0.1%–2%) in order to ascertain its lack of influence on the differentiation of mAbs. The objective was to highlight discrimination by comparing three concentrations of Infliximab (0.5, 1.0, 1.5 mg/mL) with 1 mg/mL of Nivolumab. PCA is capable of distinguishing between individuals based on the concentration of Infliximab, with a score of 71%. Furthermore, a Partial Least-Squares Discriminant Analysis (PLS-DA) model, based on previously processed electropherograms, is effective in attributing samples to the appropriate mAb cluster, without any concentration or excipient effects.

icIEF has been explored as a product identity analytical method by Ahluwalia et al. The research group evaluated the possible challenges which can be encountered during the set-up of a product identity method for mAbs and their related products with icIEF. The work emphasises that to ensure reliable results, it is essential that the sample maintains its native form and that aggregation is avoided (Ahluwalia et al., 2018, 271)**.**

In a protocol by ProteinSimple the treatment of mAbs with carboxypeptidase B (CpB) and its challenges during the procedure is discussed. CpB cleavages specifically the C-terminal lysine residues, modifying the aspect of the charge isoforms profile. Recently, in literature it has been reported that C-terminal lysine could adversely affect mAbs complement-dependent cytotoxicity (CDC) (Van Den Bremer et al., 2015, 672-680). A desalting process before the CpB treatment is useful to avoid certain formulation components inhibiting the enzyme activity. Furthermore, it is known that several pIMs which contain arginine or lysine residues could undergo CpB digestion, causing an imprecise calibration of icIEF pIs measurements. In this published protocol, Adalimumab is used as a mAb example to demonstrate the importance of desalting before the digestion with CpB. ProteinSimple showed that in order to minimise unexpected digestion of basic pIMs, inhibiting the CpB enzyme activity, two different pathways should be followed: a cooling process at 4°C or the addition of citric acid after the CpB treatment (ProteinSimple, 2018).

In icIEF it is essential to use additives for enhancing analytes solubility. Near their pI value, proteins can aggregate or precipitate due to low solubility, affecting the reproducibility of charge profiles and generating spikes during the analysis. Additives act principally as solubilizer agents, the most common example is urea, which reduces the possibility of hydrogen bond formation, avoiding protein aggregation and precipitation and helping with their solubilization (Leng et al., 2024, 343176; Turner and Schiel, 2018, 2079–2093). In literature many research articles study the effects of urea on charge variants analysis by (i)cIEF. The presence of urea can decrease signal intensity or lead to a position shift of the main peak (Kinoshita et al., 2013, 76–83); it can also have an impact on the focusing time and voltage settings (Mack et al., 2009, 4049–4058).

**FIGURE 4:**
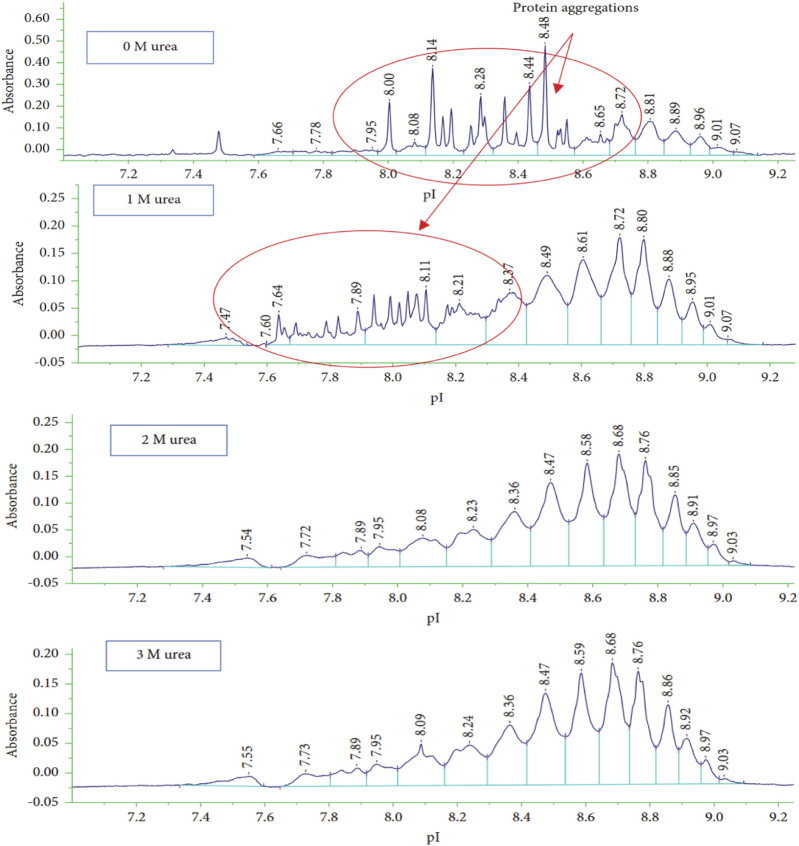
icIEF analysis of maytansinoid-antibody under different concentrations of urea (0, 1, 2, and 3 M) to study its effect on charge variants separation. The presence of the additive improves the resolution of the electropherogram, in fact 2 M of urea is chosen to obtain an optimal separation of the sample. Reproduced with permission from the ref. (Abbood, 2023, 8150143), Copyright 2024, John Wiley & Sons. (This work is licensed under CC BY-NC-ND 4.0. To view a copy of this license, visit https://creativecommons.org/licenses/by-nc-nd/4.0/).

In a recent article, the impact of different amounts of urea, ranging from 0 M to 3 M, added to a sample of ADC product, maytansinoid-humanised anti-EphA2 antibody, was evaluated (Abbood, 2023, 8150143). The results showed (Figure 4) that the addition of urea to the sample matrix improved the characterization of the charge isoforms of the sample, but also that at 1 M urea concentration spikes were generated probably due to aggregation. Charge variants profile was stable at urea concentrations above 2 M, which was chosen as the best analysis condition even if pI values were moderately decreased and the viscosity of the sample matrix was increased.

Chemicals with urea’s structural similarities, such as formamide and N-ethylurea have been reported in literature as able to enhance method robustness during icIEF analysis of difficult-to-denature proteins. Zhang et al. noted that urea and sucrose are not sufficient to obtain a robust charge profile of a typical fusion protein in icIEF assay. They suggested using formamide, since the results obtained with this additive showed that it significantly improved the robustness and repeatability of the icIEF assay (Zhang et al., 2017b, 1–7), making it a very good alternative. In a second paper, Zhang’s group studied the effect of different types of denaturing agents such as urea, formamide and non-detergent sulfobetaine mixed with taurine (NDSB-T). The results indicate that only NDSB enhances the repeatability, while NDSB-T can maintain very hydrophobic antibodies in their native condition during icIEF analysis, providing more accurate information during charge heterogeneity characterization. The novel matrix formula containing NDSB-T may be a valuable tool for proteins incompatible with conventional icIEF matrices (Zhang et al., 2017a, 13).

**FIGURE 5:**
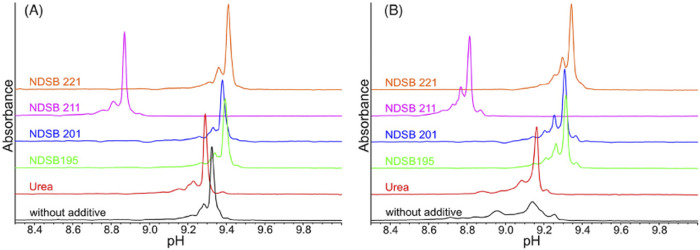
icIEF electropherograms of an IgG1 mAb **(A)** and a ZweimAb **(B)** using different types of NDSBs at 0.5 M, as additives compared to urea in concentration of 3 M or without any additive. NDSB 195 shows excellent properties, thus a suitable stabilizing additive for the specific icIEF protocol. Reproduced with permission from the ref. (Meudt et al., 2024, 1295–1306), Copyright 2024, John Wiley & Sons. (This work is licensed under CC BY-NC-ND 4.0. To view a copy of this license, visit https://creativecommons.org/licenses/by-nc-nd/4.0/).

As we have discussed above, unfolding, miss-folding, and aggregation can result in a deteriorated charge variant analysis. In a recent work (Meudt et al., 2024, 1295–1306) four different types of NDSBs, namely, NDSB 195, NDSB 201, NDSB 211, and NDSB 221, were tested as alternative stabilising additives for the analysis of mAbs and complex bispecific IgG-like mAbs (Figure 5). It was found that NDSB 195, at 500 mM concentration, exhibited excellent properties for icIEF applications. The platform provides good resolution and linearity to resolve charge species and reliably determines the pI of the considered analytes**.**

Another reagent has been reported to solubilize PEGylated proteins in a new icIEF method developed to improve the resolution of charge variants (Zhang et al., 2020, 735). Analysis of charge variants of PEGylated protein drugs is a challenging task since the PEGylation process inevitably increases the structural complexity of the conjugated protein. The addition of glycine to the icIEF matrix enabled the separation of co-migrated charge variants of PEGylated protein A. However, the addition of glycine causes significant baseline interferences reducing the assay quantitation and detection limit for basic charge variants. The issue was resolved through the addition of taurine, whose zwitterionic form competes with glycine for binding to the capillary wall. This effectively reduces matrix-induced baseline interference, allowing precise integration and quantification of basic charge variants. The precision of the newly developed method with the use of glycine-taurine sample matrix (Gly-T) was confirmed by multiple injections and multiple sample preparations of PEGylated protein A. The precision of the multiple sample preparation, evaluated by the standard deviation of the percentage peak area for acidic, main, and basic groups was very good (standard deviation 0.3%, 0.5%, and 0.3%, respectively). Linearity and accuracy studies, as well as sample stability, were carried out: linearity was confirmed with an R2 greater than 0.98; accuracy was calculated as a percentage of recovery (93.2%–109.9%, 98.0%–101.9%, and 94.6%–107.3%, respectively, for acidic group, main peak, and basic group) and resulted conform to ICH guideline. LOQ and LOD of the new icIEF method, using Gly-T matrix, were determined and confirmed to be 0.028 mg/mL and 0.008 mg/mL, respectively. Robustness and stability studies were also performed. The proposed icIEF method enables the analysis of charge variants of PEGylated proteins and antibodies and allows to capture the changes made during PEGylation and purification processes.

A non-denaturing versatile protein stabilizer (SimpleSol) was used to perform icIEF analysis of fusion proteins that are otherwise prone to aggregation or precipitation (Wu G. et al., 2022, 114505). The obtained data suggest that SimpleSol can be used as a versatile protein stabilizer in platform methods for icIEF analysis of fusion proteins as reproducible peak patterns could be acquired. The developed platform method can be used as the starting point when high resolution is required, avoiding the need for lengthy method development. The icIEF method can be applied as an identity and purity assay for fusion proteins in the biopharmaceutical industry as the results are reproducible in peak group area percentage and apparent pI determination**.**

In a recent article by Leng et al. different concentrations of urea were evaluated to analyse charge variants profile of ADCs (Leng et al., 2024, 343176). Drug-to-antibody ratio (DAR) ADCs of various values (ranging 4–8) and payload linker chemistry were considered, and it turned out that the concentration of urea gave different results depending on DAR value and payload linker. For DAR = 8 ADC with a payload-linker containing a PEG subunit the addition of 3 M or 5 M urea into the sample led to poor separation while 8 M of urea showed an increased number of peaks, generating fragments favoured by a denaturing environment. Thus, SimpleSol, a ProteinSimple additive used to solubilize proteins, was studied finding an enhanced separation of the profile with the addition of 40% or 50% of SimpleSol. The research demonstrated how SimpleSol, which is a non-ionic surfactant, is more effective in the interaction with PEG-containing linker, avoiding possible precipitation and or aggregation forms.

We can summarise that in icIEF analysis, urea is normally used to improve the repeatability of charge variants separations in proteins and antibodies and to measure more stable pI value, however sometimes other additives should be considered, regarding the type of biotherapeutic under analysis.

### 2.3 Carrier ampholytes

Carrier ampholytes (CAs) are essential components in IEF techniques. They are added to samples to generate a stable pH gradient into the capillary, enabling analytes separation based on their pI. Usually, CAs are aliphatic oligo-amino oligo-carboxylic acid molecules of different lengths and or branching (200–1,200 Da) and their electrophoretic properties differ in base to their supplier (Righetti, 1983; Righetti et al., 2007, 3799–3810; Kristl et al., 2014)**.** CAs have been marketed under trade names such as Pharmalyte, Servalyt, and AESlyte. The quality of an icIEF analysis for biotherapeutics charge variants evaluation is highly dependent on the type of carrier ampholytes utilised in terms of baseline signal, pH gradient linearity and pI measurement (Kwok et al., 2022, 2)**.**

Commonly, CAs are added in total concentrations between 2% and 8% to the samples and broad-range CAs – such as Pharmalytes 3-10, can be mixed with narrow-range ones to improve resolution. Moreover, the total concentration of Pharmalytes, as well as their assortment, can greatly influence the focusing time required for proper separation of the analytes. Thus, careful research for the best CA brand selection, the right amounts and the reciprocal ratios of CAs is necessary to achieve the best compromise in establishing the best pH gradient along which the analytes charge variants can be separated (Michels et al., 2012, 5380–5386). An important aspect to be considered in the selection of CA is the CA-specific background during the analysis. In fact, it was observed that when CAs are at a concentration above 3%-4%, the background noise of the analysis increases, influencing peak integration from baseline fluctuations. The problem occurs with both UV and fluorescent detection.

AESlytes are CAs developed for the high-resolution and enhanced characterization of complex protein therapeutics such as BsAbs, viral and fusion proteins and ADCs which demonstrate a reduction in the background noise (AES Advanced Electrophoresis Solutions Ltd, 2016)**.** This type of CAs was used in a research article by Kwok et al. aimed at studying the charge variants profiles of different commercial fusion proteins and biosimilars with high repeatability. Furthermore, narrow-range pH AESlytes were considered during icIEF-MS analysis to optimise the resolution and to obtain more reliable and accurate charge variants profiles (Kwok et al., 2022, 2).

Pharmalytes were used in a research article by Abbood to evaluate maytansinoid-antibody charge isoforms. In this study it was observed that the charge variants of the sample (pIs ranging between 7.5 and 9.0) shifted simultaneously with the calibration pI marker 9.50, in a sample mixture of 4% broad range 3–10 Pharmalyte. With the addition of the narrow-range 8-10.5 Pharmalyte (reciprocal ratio 1:1), the maytansinoid-antibody charge variants were differentiated from the pI marker 9.50 (Abbood, 2023, 8150143).

Besides Pharmalytes and AESlytes, Servalyts are another type of CAs used. Servalytes have been used for the charge heterogeneity characterization of fusion protein therapeutics. An icIEF platform method was developed for fusion proteins with pI values ranging from four to 8 (Wu G. et al., 2022, 114505). In this work, a wide pH range ampholyte, Servalyt 2–9 was used. The resolution of some fusion proteins was improved by the addition, into the preexisting carrier ampholytes mix of the platform method, of supplemental carrier ampholytes tailored for that molecule’s pI**.** In a more recent research article by Leng et al., Servalytes demonstrated to favour electrophoretic separation of high DAR ADCs charge variants, due to their sulfonate groups (Leng et al., 2024, 343176). A combination of 1% Servalyt 2%–11% and 3% Servalyt 9–11 was studied, and a better separation of the profile was obtained. However, the ADC-4 main peak showed a pI around 8.5–8.6, out of the linear pH gradient range. Servalyt 9–11 acts as a basic spacer, shifting the charge variants profile to the acidic pH range. The study demonstrated that narrow-range Servalytes are required to increase the pH gradient and achieve a linear pH gradient for the separation. Lastly, Leng et al. evidenced how the combination of Servalytes and SimpleSol allows a better separation of ADC charge variants, with charge masking effect from the payload-linker and the high DAR.

The important role of CAs was demonstrated in the development of an icIEF method for charge heterogeneity characterization of therapeutic mAbs and BsAb with pI values ranging from 6 to 10, a variety of different broad- and narrow-range ampholytes and combinations thereof were investigated. A CAs combination of Pharmalyte 5–8 and Pharmalyte 8–10.5 showed a highly linear pH gradient and covered a suitable pH range. The article also reported the advantages and disadvantages of CAs covering different pH ranges and obtained from different manufacturers (Meudt et al., 2024, 1295–1306).

It can be stated that narrower ampholytes (∆pH < 1.0) usually can increase peak resolution, thus leading to a better separation of charge variants. This was the case of PEGylated proteins icIEF analysis, where the use of narrow range ampholytes (Pharmalyte 4–6.5 and Pharmalyte 5–8) allowed a slightly better separation of charge variants but was not enough to resolve the broad co-migrating peaks due to the masking effect of PEG chain surrounding proteins. Thus, a novel icIEF matrix (Gly-T) provides an excellent solution for charge variant analysis of PEGylated proteins (Zhang et al., 2020, 735).

All these studies showed that optimal CAs should guarantee good linearity of the pH gradient in a wide pH range, low protein interactions, reproducible protein separation, low UV absorbance in order to minimize electropherogram background noise, low background noise to obtain high sensitivity during icIEF-MS analysis.

**FIGURE 6:**
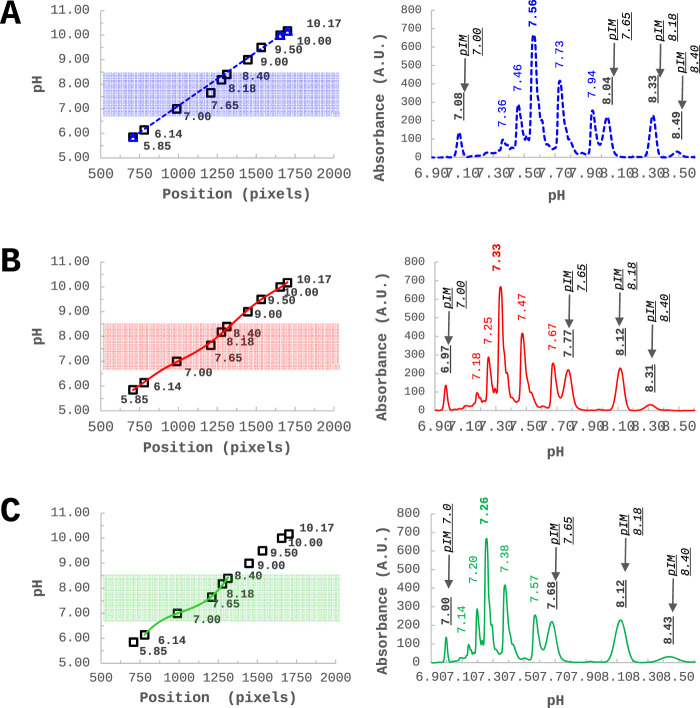
Infliximab - charge variants characterization through icIEF - analysed together with 10 pIMs, along the pH gradient. **(A)** Commonly used approach of linear calibration using three external pIMs as internal calibrators. **(B)** A non-linear calibration curve using 10 pIMs. **(C)** A non-linear calibration curve optimized in a narrow window by using five pIMs out of 10. Reproduced with permission from the ref. (Belfiore et al., 2024, 28087), Copyright 2024, Springer Nature Limited. (This work is licensed under CC BY-NC-ND 4.0. To view a copy of this license, visit https://creativecommons.org/licenses/by-nc-nd/4.0/).

Regardless of the type of ampholytes used, in (i)cIEF techniques pI values of the analyte are calculated after internal calibration. This calibration is commonly obtained with two or three pIMs, flanking the analyte, assuming a linear dependence throughout the pH gradient between pI values and migration time (cIEF) or position (in pixels) along the capillary (icIEF). In this context, Belfiore and Ascione et al. shared their experience with the ProteinSimple-Maurice™ apparatus and proposed an innovative calibration approach for iciEF in order to obtain more reliable and objective (close to theoretical) pI measurements, thus exploring the concept of univocal charge identity (Belfiore et al., 2024, 28087). The proposed new calibration approach may help to reconcile discrepancies in the pI obtained for the same sample from different devices. The assumption of a linear calibration curve, currently enforced by both icIEF and cIEF analysis software, introduces unpredictable errors in the expected pI values, across the pH gradient, despite Pearson’s determination coefficient is very close to one. To address this issue, they proposed to use a non-linear regression approach to enable recalibration of the data, as shown in Figure 6. Importantly, while assuming a linear calibration implies a constant resolution over the pH gradient, the non-linear regression reveals an actual non-homogeneous resolution along the gradient. Accordingly, they demonstrated the possibility of investigating the resolution power across the entire capillary to identify the optimal focusing conditions and CAs combination for a specific purpose.

The approach described above offers intriguing insights and aims to make possible the objective estimation of pI. The state-of-the-art of currently available softwares integrated in (i)ciEF instruments renders the above approach not yet applicable to routine control activities. At the moment, the high-level standardization effort of Ph. Eur. is recognized, delivering reliable reference for the application of (i)cIEF methodology to the control of biopharmaceuticals (2.5.44. Capillary Isoelectric Focusing for Recombinant Therapeutic Monoclonal Antibodies, 2023).

### 2.4 Anodic and cathodic stabilizers

In icIEF the analyte charge variants are focused along the desired pH region. Furthermore, a phenomenon called cathodic or anodic drift (loss of basic and acidic CAs) could occur during the focalization process, negatively impacting repeatability and reproducibility (Tardif et al., 2023, 124633)**.** Anodic and cathodic stabilizers, also known as sacrificial agents or spacers, act as buffering zones at the ends of the capillary, compressing and stabilising the pH gradient.

The main characteristic of a cathodic stabilizer is to have a pI value below the catholyte solution but above pH 10 (Kristl et al., 2014). L-Arg is an amphoteric amino acid (pI ∼ 10.76), known to suppress EOF in capillary electrophoresis and to stabilise the cathodic zone of the capillary (Kristl and Stutz, 2014, 148). Commonly, in icIEF technique L-Arg is used at concentrations ranging from 5 to 10 mM. When a narrow-range CA is added to the sample preparation, an anodic stabilizer should also be used. The main propriety is to possess a pI value above the anolyte solution but below pH 3. The most used anodic spacer is iminodiacetic acid (IDA) which is a dicarboxylic acid amine that has a pI value around 2.2 (Kristl et al., 2014) and is used at concentrations around 5-10 mM. These sacrificial agents protect CAs from leaving the capillary and avoid the analytes loss during the separation. The right quantity of these reagents must be optimised to ensure no interference as high concentrations could impact the total resolution (Kristl and Stutz, 2014, 148; Kristl et al., 2014; Kahle and Wätzig, 2018, 2492).

There are other sacrificial agents known in literature such as tetramethylethylenediamine (TEMED), which acts as a cathodic stabilizer because it is a highly basic organic compound. Another anodic stabilizer is Serine-D which has a pI of around 5.68 (Mohan and Lee, 2002, 271–276). In an article by Tardif et al. different concentrations (ranging from 0% to 5%) of those stabilizers were tested to demonstrate their efficiency. This work concluded that to prevent a good separation of the analytes with a good resolution, TEMED and Serine-D have to be added into the sample preparation respectively in concentrations of 1.2% and 2.4% (Tardif et al., 2023, 124633)**.** The effect of anodic and cathodic stabilizers on icIEF analysis is significant. Indeed, in a research article by Kahle et al. which aims to gain a comprehensive understanding of icIEF technique through the application of a DoE approach, three concentrations of L-Arg (0, 5, and 10 mM) have been studied (Kahle et al., 2019, 2382–2389). It was observed that by increasing L-Arg concentrations, the resolution decreases probably due to the correlation to a reduced separation length into the capillary. The high quantity of L-Arg is accumulated at the cathodic extremity of the capillary, causing a compressed pH gradient. L-Arg significantly influences resolution power, peak position, and peak count, but it does not affect the apparent pI measured.

### 2.5 Detection mode

**FIGURE 7:**
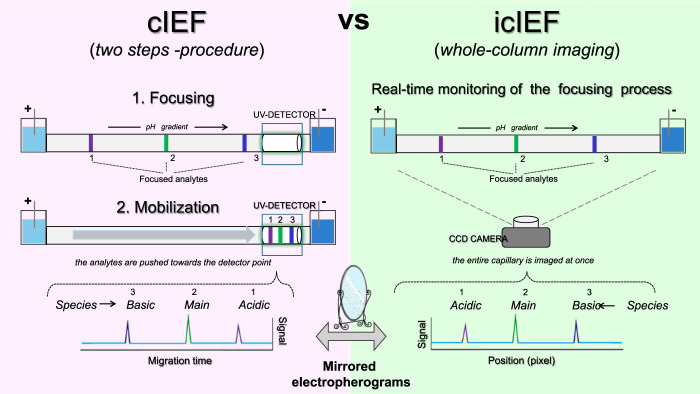
Schematic representation of the key technical differences between cIEF and icIEF, regarding data acquisition and resulting electropherograms.

The key distinction between icIEF and traditional cIEF lies in the detection step. icIEF detection method results in a significant reduction in method development time while maintaining the established advantages of high resolution, high throughput and minimal solvent consumption. In conventional cIEF, following the focusing of protein isoforms at their respective isoelectric points in a long capillary (20-60 cm), the so-called mobilisation step is required, whereby the separated species are mobilised towards the detection point, typically located at one end of the capillary. However, the mobilisation step, which is typically conducted chemically or by controlled pressure, can result in several limitations, including prolonged analysis time, distortion of the pH gradient and even occasional uneven resolution as a consequence of the unequal speed of mobilisation (Mao and Pawliszyn, 1999, 93–110). The aforementioned issues are circumvented by icIEF. In fact, in icIEF technique, the separation process occurs within a capillary of a relatively short length (4-5 cm) that is stabilised within a cartridge, while a CCD camera is positioned to capture images of the entire capillary structure. This configuration enables the real-time detection of the target protein within the capillary, thereby facilitating a markedly higher analytical speed. The two different modes of detection are reflected in a different visualization of the separated species (Figure 7). In cIEF, the more basic variants will appear first in the electropherogram since they pass through the detector first; in this system, the detected signals are displayed as a function of migration time, with the basic and acidic variants to the left and right of the main species respectively (Figure 7, left). Instead in icIEF, the entire capillary is imaged, and the peaks are plotted as a function of their position in pixels along the capillary, thus resulting in an electropherogram in which the acid species are displayed on the left and the basic species are on the right (Figure 7, right). In practical terms, apart from the anticipated similarity in shape, the arrangement of the peaks in the electropherograms of the two systems will be specular (Ascione et al., 2024, 2313737).

As previously stated, the latest generation of icIEF instruments generally guarantees high sensitivity, which is an essential prerequisite for detecting low abundance variants; however, the power level of this attribute is largely dependent on the technology used for detection. Absorption imaging detection is the most practical at present due to its quantitative ability and universal characteristics, even if various types of imaging detectors have been developed including refractive index gradient, and laser-induced fiuorescence (LIF). Usually, icIEF technique is based on protein absorption at 280 nm but nowadays instruments add native induced fluorescence (NIF) detection around 350 nm (Li et al., 2021, 462043) to enhance its capabilities. The detection process by native fluorescence is achieved through the measurement of the fluorescence emission of the aromatic group of tryptophan, a naturally fluorescent amino acid. Since this is a label-free detection, baselines are highly cleaner and less sensitive to CAs interference, as these species do not fluoresce between 320 and 450 nm. Furthermore, as stated in a ProteinSimple Application Note this sensitivity allows to avoid sample concentration or desalting, reducing sample preparation time. As outlined in this Application Note, working with native fluorescence would allow to reduce or completely remove urea during sample preparation (Improving Charge Variant, 2000). The Maurice-ProteinSimple instrument introduced a fluorescence detection filter at 458 nm which is particularly useful for ADC characterization. In fact, as it is reported in literature, if an enhanced fluorescence intensity is observed at 458 ± 30 nm, ADC samples might be analysed by icIEF fluorescence at 458 nm. This detection enables the quantification of the conjugated drug to be conducted independently of the antibody, for each charge variant. In a study case presented in a ProteinSimple Protocol, Alexa^®^ 350 fluorescent dye was conjugated at different dye-to-protein molar ratios to simulate different DARs (ProteinSimple, 2019). This study evidenced how the fluorescent conjugate can be imaged as a standalone entity, independent of the protein signal. Furthermore, the dye and antibody peaks can be attributed by overlapping the absorption and fluorescence profiles. As reported in an excellent research article by Li et al. icIEF-UV fluorescence is an auspicious technique even to characterise recombinant human ErythroPOietin (rhEPO) charge variants in DPs, without sample treatment (Li et al., 2021, 462043). The sensitivity of the combination of UV and native fluorescence was sufficient to detect low concentrations of rhEPO, while maintaining the separation power of the icIEF technique which allows to avoid interference from excipients. A method validation was performed on a commercial DP sample, following ICH guidelines, and demonstrated a 100-fold higher sensitivity in comparison to icIEF-UV but also to CZE-UV techniques.

Simultaneous monitoring of the capillary using both absorbance and fluorescence detection has been used during the analysis of fixed-dose combination (FDC) products containing different mAbs (Candreva et al., 2022, 1701–1709). It was observed that UV absorbance is ideal for analysing the charge isoforms of the high-concentration component, while native fluorescence is suited for detecting the variants of the low-concentration component.

Furthermore, as suggested by Belfiore and Ascione et al. modern icIEF devices’ capability to measure signals in both absorbance and fluorescence modes, can be exploited in order to strengthen the calibration by using multiple pIMs as internal calibrators (Belfiore et al., 2024, 28087). Assuming that an ever-widening range of differently visible pIMs (in one channel and in both) will soon be available on the market, this would make it possible to obtain a kind of independent ‘two-channel electropherogram’, without any interference for sample analysis: in other words, an optimal internal calibration curve and a clean-molecule electropherogram would be obtained simultaneously for each injection.

## 3 icIEF-MS: a novel orthogonal approach

In addition to a charge variant profiling obtained with icIEF technique coupled to UV and fluorescent detectors, mass spectrometry (MS) analyses can be also desirable to acquire identification of these charged species. icIEF-MS approach can offer significant potential in the biopharmaceutical sector, where charge variant analysis and peak identification are essential for research and development activities, including mAb-based drugs screening, purification process optimization, formulation studies, stability assessments, QC testing, and Investigational New Drug (IND)-enabling studies (Mack et al., 2019, 3084).

**FIGURE 8:**
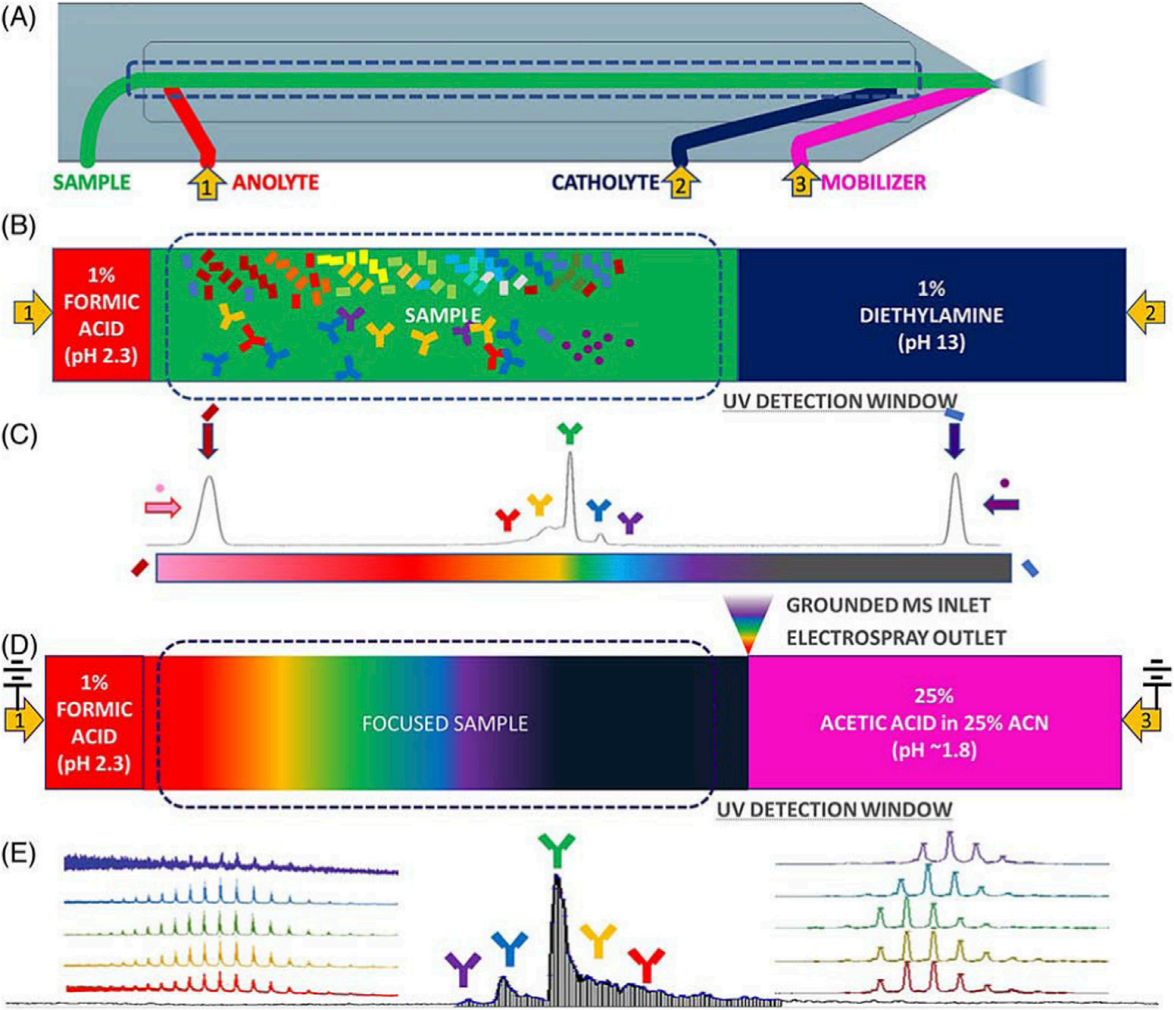
Coupled icIEF-MS workflow for the analysis of therapeutic intact protein. **(A)** Scheme of the microfluidic separation and ionization chip with electrospray outlet, inlets for solvents, and electrodes at anode, cathode, and mobilizer. **(B)** Path of the separation phase (anolyte, sample and catholyte solutions). **(C)** Focused sample and pIMs electropherogram (UV ABS). **(D)** Scheme of the initiation of mobilization and ESI of the separated sample. **(E)** Time-resolved base peak intensity plot with inset normalized raw and deconvoluted mass spectra. Reproduced with permission from the ref. (He et al., 2022, 1215), Copyright 2024, Springer Nature Limited. (This work is licensed under CC BY-NC-ND 4.0. To view a copy of this license, visit https://creativecommons.org/licenses/by-nc-nd/4.0/).

icIEF-MS analysis can be performed through offline fractionation, where collected fractions are processed with MS-compatible materials, before being introduced into the MS, for detailed and enhanced characterization. The advanced preparative icIEF system enables the simultaneous isolation and analysis of specific protein charge variants. Additionally, it can be directly integrated with MS, providing fractionated protein samples for immediate characterization (Maráková et al., 2023, e2300244). The newly directly coupled icIEF-MS technique represents a significant advancement in the field of high-resolution separation of therapeutic charge variants, enabling the quantification and identification of their individual components and the interpretation of their structural differences. This integrated method (Figure 8) is useful and adaptable for characterizing various biotherapeutics, such as fusion proteins, bispecific antibodies (BsAbs), and antibody-drug conjugates (ADCs) (He et al., 2022, 1215).

A recent study by Wu et al. introduced a novel integrated approach combining icIEF-MS for the analysis of a set of therapeutic mAb charge variants (Wu et al., 2023, 2548–2560). In this work, both icIEF-MS and strong cation exchange-MS (SCX-MS) techniques were optimised to characterise charge heterogeneity, with a particular focus on comparing their performance through methodological validation. The results demonstrated that, while SCX-MS offered higher throughput, icIEF-MS performed higher sensitivity, reduced carryover, precise protein identification, and enhanced resolution in protein separation. However, despite its advantages, icIEF-MS presents several challenges in the analysis of protein charge variants. The study highlighted issues with repeatability, complex and often trial-and-error optimization processes, and difficulties related to compatibility with MS ion sources. Notably, the chip-based icIEF-MS technique relies on chemical mobilisation for MS detection, which can induce instability in the pH gradient, leading to reduced reproducibility. The analytical platform developed in this study was thoroughly validated across sensitivity, repeatability, carryover effects, and capillary lifespan, ensuring robust and consistent results The same research group aimed to study fusion proteins’ charge isoforms with a dedicated analytical platform (Wu G. et al., 2022, 114505). This work presented a novel approach for the comprehensive characterization of etanercept analogues in QC and manufacturing processes, utilising AESlyte and allowing an enhanced resolution also of the complex glycosylation patterns. The study compared icIEF profiles with the output of HPLC-HRMS peptide mapping and PTMs analysis, finding an agreement between the two techniques which could be used for the production and QC of fusion proteins.

**FIGURE 9:**
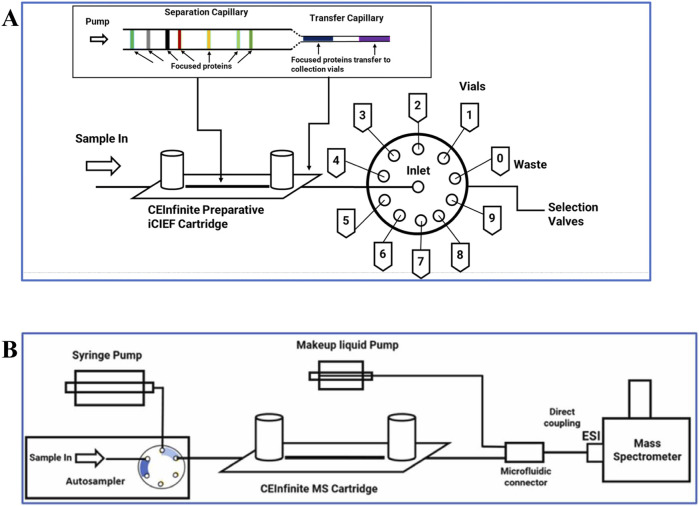
Configuration of **(A)** an icIEF-based fraction collection model by preparative icIEF and **(B)** online icIEF-HRMS. Reproduced with permission from the ref. (Kwok et al., 2023b, 411–418), Copyright 2024, ^©^ The Royal Society of Chemistry. (This work is licensed under CC BY-NC-ND 4.0. To view a copy of this license, visit https://creativecommons.org/licenses/by-nc-nd/4.0/).

Native MS (nMS) has recently emerged for the analysis of large biomolecules, allowing the characterization of protein complexes and their structural features in a solution environment that closely mimics their native state. nMS allows the study of noncovalent protein assemblies while retaining their biological relevance. Zhang’s research group developed a whole workflow of icIEF-MS strategy for a rapid fingerprint of intact proteins which demonstrated to be reliable and accurate and provided a comprehensive and innovative technology for protein drug QC monitoring and in-depth characterization (Zhang et al., 2023a, 114961). In another research article by the same group, the whole icIEF-MS workflow for protein heterogeneity was performed within 45 min. Moreover, the developed icIEF-MS configuration was capable of adapting to an icIEF-based fraction collection model (Figure 9), thereby enabling the analyst to carry out supplementary in-depth characterisation techniques, such as peptide mapping by HPLC and LC-MS/MS. The established methodology was highly sensitive and accurate, during the heterogeneity evaluation of mAb and ADC. icIEF-HRMS can provide a promising, accurate and rapid strategy for the differentiation and identification of protein charged variants, supporting the rapid growth and need of biotherapeutics (Zhang et al., 2023b, 115178). In a more recent paper, Zhang et al. used the icIEF-MS approach to analyse the charge variants of cysteine-linked ADCs, which are crucial during drug development. Two distinct cysteine-linked ADCs were examined: Polatuzumab vedotin, a novel ADC with promising therapeutic applications, and Brentuximab vedotin, the first FDA-approved ADC of this kind. One of the key achievements in this research work is the development of an optimised icIEF buffer system, which ensures the native conformation of cysteine-linked ADCs during the analysis, enabling a more accurate charge variant profiling (Zhang et al., 2024).

Furthermore, Wu et al. evaluated an advanced online coupling of icIEF-MS under near-native conditions, specifically designed for the in-depth characterization of cysteine-linked ADCs (Wu et al., 2024c, 465353). The characterization of cysteine-linked ADCs presents significant challenges due to the presence of interchain disulfide bonds that are reduced during payload conjugation, as well as the non-covalent interactions between the antibody light and heavy chains. However, despite the potential advantages of icIEF-MS, characterising cysteine-linked ADCs remains difficult because maintaining the integrity of the conjugated structure for intact MS analysis requires native conditions, and the associated parameters must be carefully optimised. By keeping the cysteine-linked ADCs in their near-native state, the developed method enabled high-resolution MS detection without compromising the integrity of the conjugated structure. In an article by Mack et al. the icIEF-MS technique was evaluated for the characterization of Trastuzumab charge variants, in its intact state. In this study, five major glycoforms in a single assay of 15 min were detected and 33 distinct molecular species were separated by the icIEF-MS technique. This allowed the identification and monitoring of several CQAs in a single exhaustive analysis (Mack et al., 2019, 3084).

**FIGURE 10:**
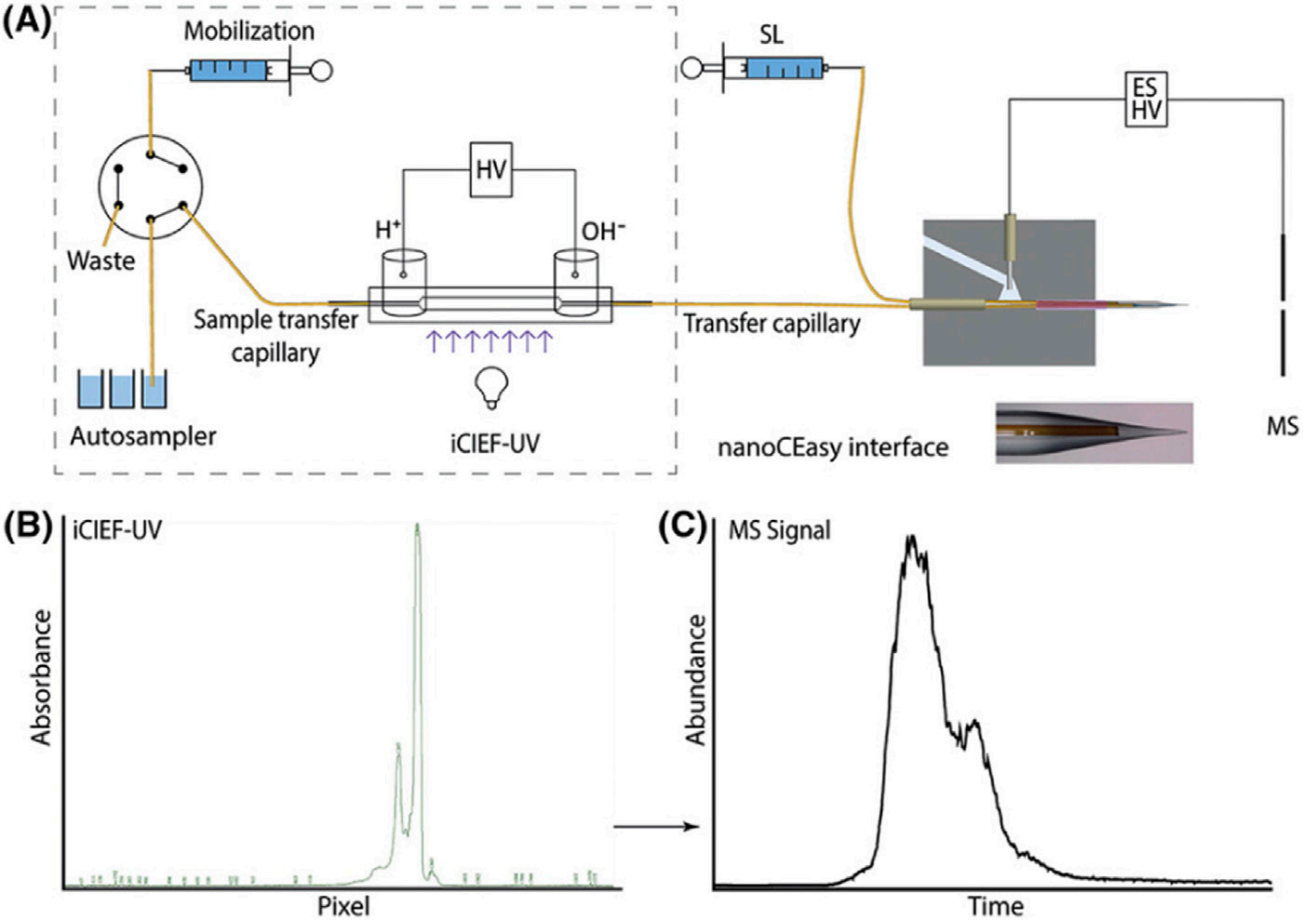
**(A)** Scheme of the CEInfinite with icIEF cartridge connected with the nanoCEasy ESI–MS interface. **(B)** iCIEF–UV profile of 2 mg/mL Trastuzumab. **(C)** Base peak electropherogram m/z 1500–4000 of Trastuzumab with Orbitrap Fusion Lumos. Reproduced with permission from the ref. (Schlecht et al., 2022, 540), Copyright 2024, John Wiley & Sons. (This work is licensed under CC BY-NC-ND 4.0. To view a copy of this license, visit https://creativecommons.org/licenses/by-nc-nd/4.0/).

Recent advancements have introduced new microfluidic chip-based icIEF systems directly coupled with MS, further improving the precision and efficiency of charge variant peak identification. In an article by He et al. the comparability of the measured pI values and the relative charge isoforms distribution between icIEF-MS technique and a routinely utilised methodology is demonstrated (He et al., 2022, 1215). Different IgG mAbs subclasses along a pI range between 7.3 and 9.0 were evaluated. Thanks to the high-performance icIEF-MS system, low abundance PTMs were also detected. In this study acidic and basic shifts were noticed, corresponding to additions of sialic acids and unprocessed lysine residues, respectively, due to a primary structural characterization of the IgG2 mAb subclass. The icIEF-MS analysis was able to detect particularly low abundance glycoforms (e.g., G0, G0F-GlcNAc)**.** Moreover, Schlecht’s working group set up a direct icIEF-MS coupling procedure, via nanoCEasy interface, enabling a successful application for narrow charge variant profile of Trastuzumab (Figure 10). The study investigated crucial parameters (e.g., sample concentration, ampholytes concentration, etc.) which could affect the charge variants profiling. The obtained parameter values were a functional starting point and needed to be optimised depending on the MS system used. To avoid loss of peaks during the detection, low mobilisation flow rate and ampholyte concentration >3% are required (Schlecht et al., 2022, 540).

In a research article by Ostrowski et al. a novel method for the rapid, multi-attribute characterization of BsAbs using an enhanced microfluidic chip-based integrated icIEF-MS technology. The improved approach utilizes a nebulization gas at the electrospray tip of the microchip during the delivery into the MS (Ostrowski et al., 2022, 378). This icIEF-MS platform developed allows a direct and simultaneous analysis of multiple CQAs of BsAbs and the characterization of the charge variants in their native state with a high-resolution power. Furthermore, the use of a microfluidic chip system allowed a faster and more efficient analytical process, reducing sample volume and improving the throughput.

Recently, the already cited Wu’s research group has carried out a lot of in-depth analysis on icIEF-MS to characterise SARS-CoV-2 recombinant vaccine, combined with different separative analytical techniques (Wu et al., 2024b, 342349). Due to the higher complexity of the vaccine, compared to mAbs, Wu’s research group decided to eliminate the glycans by using PNGaseF. The obtained results demonstrated the crucial role of high-resolution CAs during icIEF-MS separation and its importance to obtain a fast identify of the recombinant vaccine complexity, like those used in the fight against COVID-19. This work emphasises the necessity of high-resolution analytical methods for ensuring the consistency and quality of vaccines.

## 4 icIEF relevance in biopharmaceutical context: advanced applications

Industry users apply icIEF methods for the verification of identity and purity at the level of characterization and drug substance (DS)/DP release. This technique is an optimal tool to monitor the heterogeneity profile of mAb products, which provides insights into the purity of the molecule and, in combination with peptide mapping to detect primary sequence and potency testing to address functional activity, deliver a comprehensive picture of product identity.

A research article demonstrated the challenges that can come across while establishing a product identity method for a mAb (pI∼7.9) and 10 in-house mAbs products with close pI range (pI∼7.0–8.5) using icIEF method (Ahluwalia et al., 2018, 271). mAbs were analysed by icIEF method under native, enzymatic and reduced conditions and a unique three-point identity criteria tool (visual comparison, pI of individual peaks and ΔpIs) was applied to distinguish mAb1 from the other in-house mAbs. A reduction approach followed by icIEF showed higher potential for establishing identity for mAb product as compared to native and enzymatic digestion approach.

An icIEF analytical method has been set up with the aim of obtaining the characterization of a novel humanised anti-EphA2 antibody conjugated to a maytansine derivative charge variant (Abbood, 2023, 8150143). A validation protocol of the optimised icIEF method was carried out and a good inter-day repeatability with the following RSD values: <1% (pI), <8% (% peak area), and 7% (total peak areas) was demonstrated. Indeed, this optimised icIEF method has been applied to evaluate the quality of a discovery batch of maytansinoid-mAb, in comparison to its naked antibody.

**FIGURE 11:**
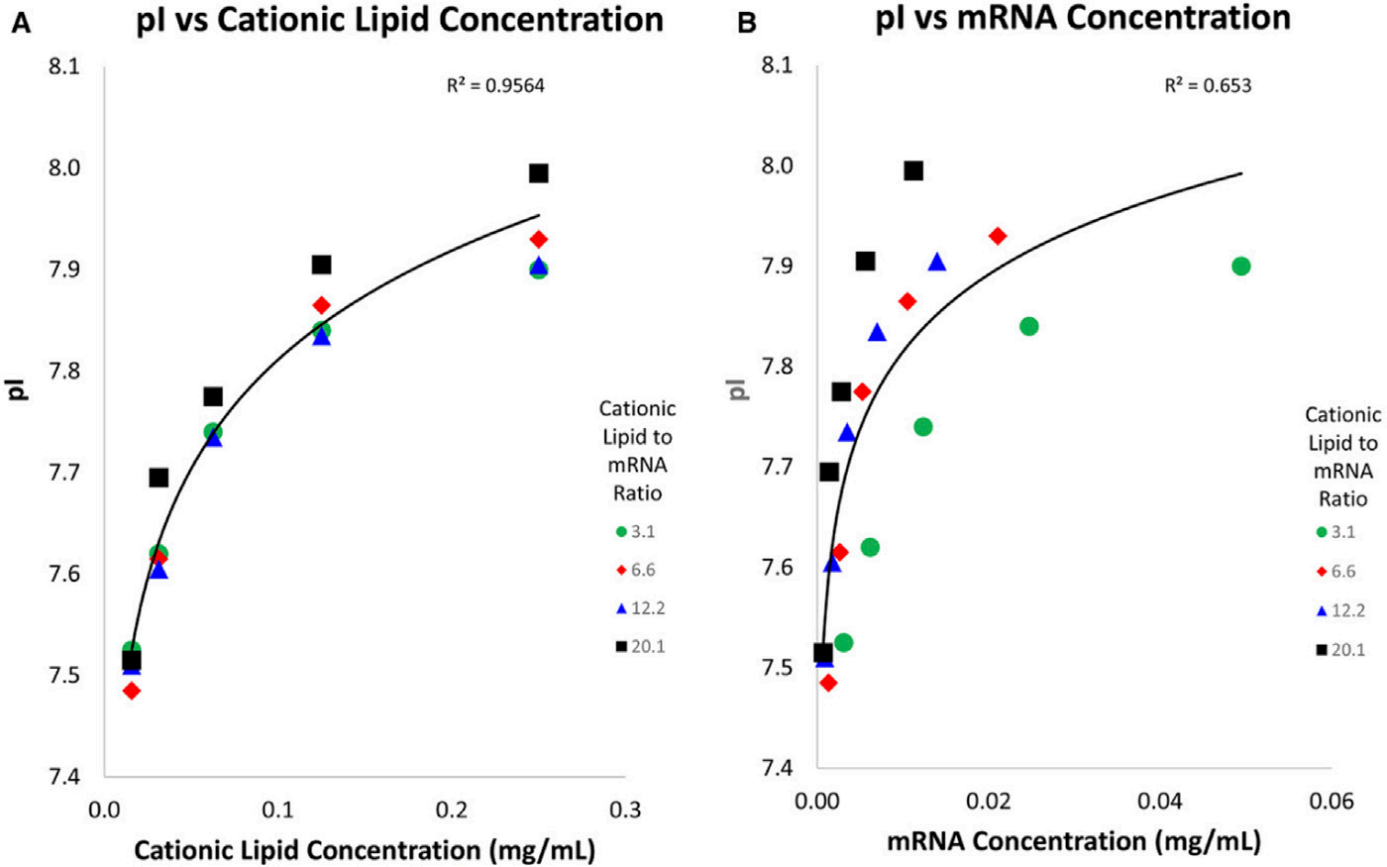
Graphics of LNP pI against cationic lipids **(A)** and mRNA concentration **(B)**: different LNP batches containing different cationic lipids to mRNA ratios. The apparent pI was found to have a strong correlation to cationic lipids concentration but a weaker correlation to mRNA concentration. Reproduced with permission from the ref. (Loughney et al., 2019, 2602–2609), Copyright 2024, John Wiley & Sons. (This work is licensed under CC BY-NC-ND 4.0. To view a copy of this license, visit https://creativecommons.org/licenses/by-nc-nd/4.0/).

In addition, icIEF can be employed to assess the surface charge of lipid nanoparticle (LNP)-based mRNA vaccines (Loughney et al., 2019, 2602–2609). This technique is capable of differentiating the pI of LNPs that contain one or more types of ionizable lipids, aiding in the verification of LNP identity during manufacturing processes. As a quantitative method, icIEF also provides insights into the stability of LNPs, making it applicable for both process optimization and formulation development of mRNA vaccines. Furthermore, it can distinguish between LNPs that incorporate various cationic lipids, serving as a useful tool for confirming the identity but also to study the correlation between LNP apparent pI and cationic lipids and mRNA concentrations. Four different batches of LNP containing different cationic lipid to mRNA ratios were subjected to icIEF analysis (Figure 11). The results indicate that the cationic lipid is situated on the surface of the LNP, while the mRNA is located within. This is a reasonable assumption, given that the LNP fulfils the function of a hydrophobic protective barrier, thereby protecting the mRNA.

Two distinct icIEF approaches have been developed, as reported in the literature, to evaluate the quality and stability of mRNA vaccines, particularly those encapsulated in LNPs. These methods are effective for characterising the stability of mRNA-loaded LNPs under different conditions, such as varied storage temperatures, freeze-thaw cycles, and lipid compositions, and are capable of detecting batch-to-batch variability. Overall, icIEF analysis has proven to be highly suitable for characterising mRNA vaccines. It has been an essential analytical tool during the COVID-19 pandemic and will continue to play a significant role in the development of future mRNA-based products (Krebs et al., 2022, 1971–1983). The assessment of batch-to-batch consistency is of pivotal importance in the context of regulatory compliance. Consequently, other research groups developed robust and reproducible methodologies for the fulfilment of these tasks. Indeed, Sutton et al. developed and optimised an icIEF method for an in-house recombinant humanised IgG1k mAb expressed in Chinese Hamster Ovary (CHO) cells (Sutton et al., 2024, 5450–5458). Its purpose was to assess different lots consistency in terms of pIs and peak area percentage of main peak, acidic and basic isoforms. Furthermore, the method was employed to analyse different batches of Herceptin (Trastuzumab) produced in the European Union (EU) and the United States (US).

Recently, an icIEF method has been developed and tailored for the characterization of high DAR ADCs charge heterogeneity. The study showed how this optimised icIEF condition is able to quantify charge variants with higher precision and resolution but also improved sensitivity. The method lets to differentiate contributions from the protein and payload-linker (Leng et al., 2024, 343176)**.**

icIEF has been investigated as a potential multi-attribute method (MAM) for a detailed characterization and QC of BsAbs in a recent article (Wu et al., 2024a). This work demonstrated that icIEF is able, detecting and quantifying BsAbs charge isoforms, to provide detailed insights on their biophysical characteristics, providing an identity, purity and impurity assay. The method was validated and the native fluorescence detection was used to achieve good sensitivity (down to 4 μg/mL LOQ). The proposed icIEF method is able to measure pI values and quantify the relative abundance of each charge isoform as well as the impurities of homodimer mAb, generated during BsAb assembly. The research group studied the use of icIEF for optimising BsAbs production conditions in order to decrease the number of unwanted heterogeneities. Furthermore, a novel workflow has recently been presented to correlate the charge isoforms of a BsAb to its function. This approach involves the use of icIEF-MS and surface plasmon resonance (SPR) techniques (Bio-Techne, n.d.). The work investigated a therapeutic BsAb, named Mosunetuzumab to determine affinity and binding kinetics of different charge species. High-purity fractions of the BsAb were collected from the icIEF instrument and examined with SPR to comprehend their affinity to ligands CD3 and CD20. This method outlines the significance of utilising advanced techniques during the development and QC of biotherapeutics.

A notable application of icIEF is its use in characterising charge isoforms of FDC products. Combination therapies involving two or more therapeutic mAbs have gained prominence in oncology and the need for accurate characterization of these complex formulations has grown. In a recent study, an optimised icIEF methodology was developed to allow simultaneous quantification of acidic and basic charge variants in FDC products containing two different mAb isotypes (IgG1 and IgG4), each with distinct pI values (Candreva et al., 2022, 1701–1709). The developed icIEF approach is a QC-friendly solution, adhering to critical acceptance criteria outlined by the ICH, including sensitivity, specificity, linearity, and repeatability. By employing a dual-detection approach, this method ensures high accuracy and reproducibility, even for complex mAb mixtures.

## 5 Conclusion

icIEF technique is an indispensable tool for protein product monitoring and is becoming the platform method of choice for analysing protein charge heterogeneity due to its high-resolution power, quantitative capabilities, robustness, fast analysis times and automation. It can be stated that icIEF is certainly a consolidated technique for monitoring the heterogeneity of the charge variants and the quality of proteins and mAb-based drugs, but the application horizons of icIEF are constantly broadening. Recently, more tailored icIEF approaches in the characterization of ADCs, BsAbs, fusion proteins and LNPs are described in literature. The analysis of more complex proteins has magnified some technical difficulties of icIEF such as protein solubility, pH gradient resolution, capillary wall adsorption and pI reproducibility. Although the method’s development is rapid, numerous parameters must be considered such as CAs, additives selection, anodic and cathodic stabilizers to optimise charge variants separation (Table 1). At present, there is a wide collection of both broad and narrow range CAs with different molecular properties that can be leveraged to optimize icIEF resolution for specific samples. Other important aspects in icIEF method optimization are sample preparation and capillary properties. For example, there are durable and specialized hydrophilic coatings (made from MC) that can reduce some issues such as bubble formation and capillary clogging, thus expanding the samples that can be analysed by icIEF.

The wide range of variables that can influence this analytical method make it a good candidate for the development of Analytical Quality by Design (AQbD) approaches, according to ICH Q14 guideline, to develop platform methods able to control charge heterogeneity of specific molecular classes (e.g., mAbs). This may be a great advantage to manufacturers, where standardization of the analysis is made initially as a platform and then transferred by reduced validation exercise to more molecules from the same class. This approach is interesting since its potential to reduce time and costs of the development phase is evident.

From the point of view of equipment, we have witnessed a notable technological development of icIEF, starting with the introduction of fluorescence detectors capable of guaranteeing greater sensitivity, followed by instruments that combines icIEF with MS, allowing a rapid identification of intact charge variants.

It can be underlined that, at present, the correct interpretation of icIEF data requires expertise. In future, applications of artificial intelligence (AI) in managing complex icIEF data sets will be expected to play an important role, particularly in understanding the impact of charged variants on product performances. All technological and digital advances will make icIEF more and more a powerful tool throughout all stages of biotherapeutic development and manufacturing, ensuring the safety and efficacy of biopharmaceutical products.
